# Analysis of factors associated with patient-reported outcome (PRO) score completion rate one year after shoulder surgeries

**DOI:** 10.1016/j.jseint.2023.08.008

**Published:** 2023-09-14

**Authors:** Paul V. Romeo, Aidan G. Papalia, Matthew G. Alben, Luilly Vargas, Joseph D. Zuckerman, Mandeep S. Virk

**Affiliations:** Division of Shoulder and Elbow Surgery, Department of Orthopedic Surgery, NYU Grossman School of Medicine, NYU Langone Orthopedic Hospital, NYU Langone Health, New York, NY, USA

**Keywords:** Response rate, Survey completion, Nonresponse bias, Patient-reported outcomes, Survey responsiveness, Patient follow-up, PROMIS

## Abstract

**Background:**

Patient-reported outcome measurements (PROMs) are important metrics for monitoring improvements following shoulder surgery. Despite the easy accessibility of electronic PROM surveys, completion rates vary, and factors predictive of survey completion for patients enrolled in medical survey follow-up after shoulder surgery remain largely unknown. The purpose of this study is to investigate survey completion rates for common shoulder procedures and identify factors predictive of PROM completion at one-year postoperatively. We hypothesize that the response rate to shoulder PROMs may vary by the shoulder procedure type after surgery.

**Methods:**

Patients undergoing total shoulder arthroplasty (TSA), rotator cuff repair (RCR), and instability surgery (Latarjet procedure [LP], and arthroscopic Bankart repair [ABR]) from 2019 to 2021 were prospectively enrolled. Each patient was administered PROM surveys via email preoperatively and at 2-weeks, 6-weeks, 3-months, 6-months, and 12-months following surgery. Demographics and socioeconomic characteristics were collected from our institutional database. The primary outcome studied was survey completion rate by procedure. Multivariable logistic regression was performed to identify factors predictive of completing 12-month follow-up.

**Results:**

A total of 514 (251 TSA, 194 RCR, and 69 instability surgery (35 LP, 34 ABR)) patients with an average age of 58 ± 15 years were included in this study. Overall, the 12-month survey completion rate for all procedures was 57.2%. TSA had the highest completion rate (64.9%), followed by RCR (52.1%), ABR (44.2%), and LP (42.9%). ABR and LP demonstrated more than a 50% drop in survey response at 2 weeks, and the RCR cohort demonstrated an increased attrition in survey response at the 6-month mark. Patients who completed the 12-month follow-up survey were older [61 ± 14 vs. 54 ± 17; *P* < .001], less frequently self-identified as Hispanic [13% vs. 23%; *P* = .009], less frequently single [32% vs. 44%; *P* = .008], and most frequently classified as the American Society of Anesthesiology [ASA] score II [65%, *P* = .001].

**Conclusion:**

Postoperative PROM survey completion rates vary significantly among commonly performed shoulder procedures during the first year after surgery. Hispanic ethnicity and younger age were all predictive of a lower propensity, and the TSA procedure is predictive of higher odds for PROM survey completion at the 12-month follow-up.

Over the last decade, patient-reported outcome measures (PROMs) have been increasingly used as an objective way to measure patient outcomes and satisfaction.^1^^,^^13^^,^^32^^,^^46^ The use of these surveys has improved our understanding of patient experiences and perspectives following surgical interventions, ultimately shedding light on the challenges they face throughout the postoperative time course. However, challenges regarding time, cost, and burden utilized for the collection of the surveys must be considered when implementing PROMs as a part of clinical practice.^1^^,^^18^^,^^22^^,^^29^^,^^37^^,^^40^^,^^41^^,^^48^^,^^49^^,^^56^

Information obtained from these surveys is highly valuable but can be limited with regards to its impact on clinical medicine due to nonresponse bias.^59^ Previous investigations have examined risk factors associated with such a bias with many concluding that males and younger patients are less likely to respond to follow-up surveys.^16^^,^^21^^,^^23^^,^^26^^,^^28^^,^^31^^,^^54^^,^^55^ Although these shed some light on the issue, their validity is limited as their data were obtained by contacting patients through insurance databases, Press-Ganey medical practice surveys, random population sampling, and nationwide databases; all universally failing to contact and consent patients for their participation prior to sending them a survey.^16^^,^^23^^,^^28^^,^^31^^,^^54^^,^^55^ This often-overlooked nuance is essential when considering the number of surveys administered nationally and internationally has continually increased leading to nonresponse because of survey fatigue, lack of interest, and “overexposure to the survey process.”^39^^,^^50^

We believe it is necessary to investigate a patient population that has consented to taking part in the survey process and understand the barriers that may be encountered limiting to their continued participation. The purpose of this study is to investigate survey completion rates for common shoulder procedures and identify factors predictive of PROM completion at one-year postoperatively.^15^^,^^17^^,^^20^^,^^35^ We hypothesize that the response rate to shoulder PROMs may vary by the shoulder procedure type after surgery.

## Methods

### Ethics

Internal institutional review board approval was granted for this study (study ID: s21-01089). All subjects provided informed consent prior to enrollment.

### Subject selection

Subjects undergoing total shoulder arthroplasty (TSA), rotator cuff repair (RCR), Latarjet procedure (LP), or arthroscopic Bankart repair (ABR) were prospectively enrolled from March 2019 to November 2021. Reflective of the literature, these four procedures were selected for this study as they were the most common shoulder procedures performed in our division. Subjects were consented for enrollment if they met all the following inclusion criteria: (1) 18 years of age or older; (2) had completed preoperative Patient-Reported Outcomes Measurement Information System (PROMIS) computer adaptive testing (CAT) version 2.0 upper extremity (P-UE), PROMIS pain interference (P-Interference), PROMIS pain intensity (P-Intensity), and American Shoulder and Elbow Surgeons (ASES) score surveys; (3) had access to a personal computer at home; and (4) demonstrated mental capacity and English proficiency necessary to provide informed consent. Patients were excluded from the study if they did not meet the inclusion criteria, declined to consent to the study, or were deceased by the 12-month follow-up survey. Subjects were divided into cohorts based on their respective procedure. Demographics and socioeconomic characteristics were collected for each patient from electronic medical record review including age, sex, marital status, ZIP code, social deprivation index, smoking status, body mass index, American Society of Anesthesiology (ASA) score, comorbidities, and race.

### Survey and data collection

Subjects enrolled in our study were asked to complete five surveys via email preoperatively and at 2-weeks, 6-weeks, 3-months, 6-months, and 12-months following surgery. All subjects completed surveys for P-UE, P-Interference, P-Intensity, and ASES for each respective time point. Surveys were administered and monitored for completion via our institutionally hosted REDCap electronic data capture software (Vanderbilt University, Nashville, TN, USA).^19^ Up to five reminder emails were sent at each postoperative time point when subjects failed to complete a survey. Patients unwilling or unable to complete email surveys were provided the option to complete surveys over the phone or during postoperative follow-up visits. As previous investigations have demonstrated, telephonic vs. email surveys display no significant differences with regards to scores.^1^^,^^6^ This was done to reduce potential sampling and response bias toward patients who were unable to complete the study via the internet.^1^^,^^2^^,^^18^

### Patient-reported outcome measures

Patients completed PROMIS, a validated assessment tool which utilizes CAT to minimize survey burden and provides standardized scores allowing an individual's scores to be compared to all test takers.^12^^,^^42^^,^^45^^,^^58^ Each subject completed P-UE CAT version 2.0, P-Interference, and P-Intensity which assess physical function, limitations in function secondary to pain, and pain severity, respectively. PROMIS scores are normalized from 0 to 100, utilizing T-scores with a population mean of 50 and standard deviation of 10.^8^ Patients were also required to complete the ASES, a validated assessment of upper extremity function which provides a composite score based upon patient responses to questions assessing limitations in function and pain severity as measured by the visual analog scale. ASES has demonstrated good correlation to PROMIS scores for various upper extremity procedures and pathologies.^5^^,^^24^^,^^27^^,^^38^^,^^51^^,^^53^

### Statistical analysis

The primary purpose of this study is to determine what shoulder procedures and factors may impact survey retention rates for each shoulder procedure at 12 months after surgery. Descriptive statistics were performed to compare patient demographics among different procedure groups. Patient characteristics were compared between those completing and those failing to complete the 12-month follow-up surveys. Procedure type, age, ASA classification, body mass index, smoking status (ever vs. never smoker), and comorbidities were entered into a multivariable logistic regression model to identify factors predictive of completing the 12-month follow-up survey. Statistical analysis was performed using Python Jupyter Notebook (Project Jupyter, New York, NY, USA). For all analyses, *P* values <.05 were considered significant.

## Results

### Demographics

The study included 514 patients (298 male, 216 female) with a mean age of 58 ± 15 years who met the inclusion criteria for this study. Evaluating by procedure, there were 251 patients (129 male, 122 female) with a mean age of 66 ± 9 years in the TSA cohort, 194 patients (113 male, 81 female) with a mean age of 58 ± 11 years in the RCR cohort, 34 (27 male, 7 female) patients with a mean age of 31 ± 15 years in the ABR cohort, and 35 patients (29 male, 6 female) with a mean age of 31 ± 14 years in the LP cohort. As expected, based on age differences, patients undergoing LP and ABR showed a higher frequency of ASA I score (*P* < .001) and a lower frequency of hyperlipidemia (*P* < .001). Comprehensive patient demographics can be seen in Table I.

**Table I:** Baseline demographics.

	Overall n = 514	ABR n = 34	LP n = 35	TSA n = 251	RCR n = 194	*P* value∗
Age, mean (SD)	58 (15)	31 (15)	31 (14)	66 (9)	58 (11)	<.001
Age group, n (%)						
<25	30 (5.8)	15 (44.1)	14 (40.0)	0 (0.0)	1 (0.5)	<.001
25-35	27 (5.3)	11 (32.4)	12 (34.3)	0 (0.0)	4 (2.1)	
35-45	30 (5.8)	2 (5.9)	4 (11.4)	7 (2.8)	17 (8.8)	
45-55	71 (13.8)	3 (8.8)	0 (0.0)	22 (8.8)	46 (23.8)	
55-65	150 (29.2)	0 (0.0)	5 (14.3)	72 (28.7)	73 (37.8)	
65+	206 (40.2)	3 (8.8)	0 (0.0)	150 (59.8)	53 (27.5)	
Sex, n (%)						
Female	216 (42.0)	7 (20.6)	6 (17.1)	122 (48.6)	81 (41.8)	<.001
Male	298 (58.0)	27 (79.4)	29 (82.9)	129 (51.4)	113 (58.2)	
BMI, mean (SD)	29.2 (6.1)	27.1(5.1)	26.6 (4.8)	29.6 (5.9)	29.4 (6.6)	.008
Smoking history, n (%)						
Ever	215 (41.8)	7 (20.6)	11 (31.4)	133 (53.0)	64 (33.0)	<.001
Never	299 (58.2)	27 (79.4)	24 (68.6)	118 (47.0)	130 (67.0)	
Race, n (%)						
African American	49 (9.5)	4 (11.8)	3 (8.6)	16 (6.4)	26 (13.4)	.001
Hispanic	84 (16.3)	5 (14.7)	7 (20.0)	28 (11.2)	44 (22.7)	
White	365 (71.0)	25 (73.5)	23 (65.7)	203 (80.9)	114 (58.8)	
Asian	16 (3.1)	0 (0.0)	2 (5.7)	4 (1.6)	10 (5.2)	
Marital status, n (%)						
Married	293 (57.0)	9 (26.5)	6 (17.1)	159 (63.3)	119 (61.3)	<.001
Single	184 (35.8)	25 (73.5)	29 (82.9)	68 (27.1)	62 (32.0)	
Divorced	37 (7.2)			24 (9.6)	13 (6.7)	
SDI, n (%)						
1	94 (18.3)	10 (29.4)	6 (17.1)	44 (17.5)	34 (17.5)	.050
2	44 (8.6)	3 (8.8)	3 (8.6)	23 (9.2)	15 (7.7)	
3	101 (19.6)	6 (17.6)	6 (17.1)	51 (20.3)	38 (19.6)	
4	144 (28.0)	5 (14.7)	10 (28.6)	79 (31.5)	50 (25.8)	
5	56 (10.9)	6 (17.6)	5 (8.6)	28 (11.2)	19 (9.8)	
6	44 (8.6)	1 (2.9)	5 (14.3)	17 (6.8)	21 (10.8)	
7	23 (4.5)	3 (8.8)	0 (0.0)	6 (2.4)	14 (7.2)	
8	3 (0.6)	0 (0.0)	0 (0.0)	3 (1.2)	0 (0.0)	
9						
10	5 (1.0)	0 (0.0)	2 (5.7)	0 (0.0)	3 (1.5)	
ASA, n (%)						
1	53 (10.3)	11 (32.4)	13 (37.1)	9 (3.6)	20 (10.3)	<.001
2	310 (60.3)	16 (47.1)	15 (42.9)	151 (60.2)	128 (66.0)	
3	146 (28.4)	7 (20.6)	7 (20.0)	88 (35.1)	44 (22.7)	
4	1 (0.2)	0 (0.0)	0 (0.0)	0 (0.0)	1 (0.5)	
Unknown	4 (0.8)	0 (0.0)	0 (0.0)	3 (1.2)	1 (0.5)	
Comorbidities						
DM, n (%)	73 (14.2)	3 (8.8)	3 (8.6)	37 (14.7)	30 (15.5)	.566
HTN, n (%)	260 (50.6)	8 (23.5)	10 (28.6)	160 (63.7)	82 (42.3)	<.001
HLD, n (%)	235 (45.7)	6 (17.6)	13 (37.1)	141 (56.2)	75 (38.7)	<.001

*ABR*, arthroscopic Bankart repair; *LP*, Latarjet procedure; *TSA*, total shoulder arthroplasty; *RCR*, rotator cuff repair; *SD*, standard deviation; *BMI*, body mass index; *SDI*, social deprivation index; *ASA*, American Society of Anesthesiology; *DM*, diabetes mellitus; *HTN*, hypertension; *HLD*, hyperlipidemia.

∗ *P* values for one-way analysis of variance (continuous) or chi-squared test or Fisher exact test (categorical). *P* values determined for comparison between ABR, LP, TSA and RCR cohorts.

### Survey response rates in first year after shoulder surgeries

Survey completion rates varied between procedures at each time point in the first postoperative year (Table II). A total of 57% (294 or 514) of all patients completed 12-month follow-up surveys with the LP cohort demonstrating the lowest survey completion rate [15/35 (43%)], while patients within the TSA cohort demonstrated the highest completion rate [163/251 (65%)].

**Table II:** Retention rate by procedure.

	Overall (n = 514)	ABR (n = 34)	LP (n = 35)	TSA (n = 251)	RCR (n = 194)	*P* value∗
2 week, n (%)	345 (67.1)	17 (50.0)	15 (42.9)	171 (68.1)	142 (73.2)	.001
6 week, n (%)	366 (71.2)	17 (50.0)	15 (42.9)	187 (74.5)	147 (75.8)	<.001
3 mo, n (%)	358 (69.6)	15 (44.1)	18 (51.4)	186 (74.1)	139 (71.6)	<.001
6 mo, n (%)	336 (65.4)	16 (47.1)	16 (45.7)	177 (70.5)	127 (65.5)	.003
12 mo, n (%)	294 (57.2)	15 (44.1)	15 (42.9)	163 (64.9)	101 (52.1)	.004

*ABR*, arthroscopic Bankart repair; *LP*, Latarjet procedure; *TSA*, total shoulder arthroplasty; *RCR*, rotator cuff repair.

∗ *P* values determined for comparison between ABR, LP, TSA and RCR cohorts.

**Figure 1:**
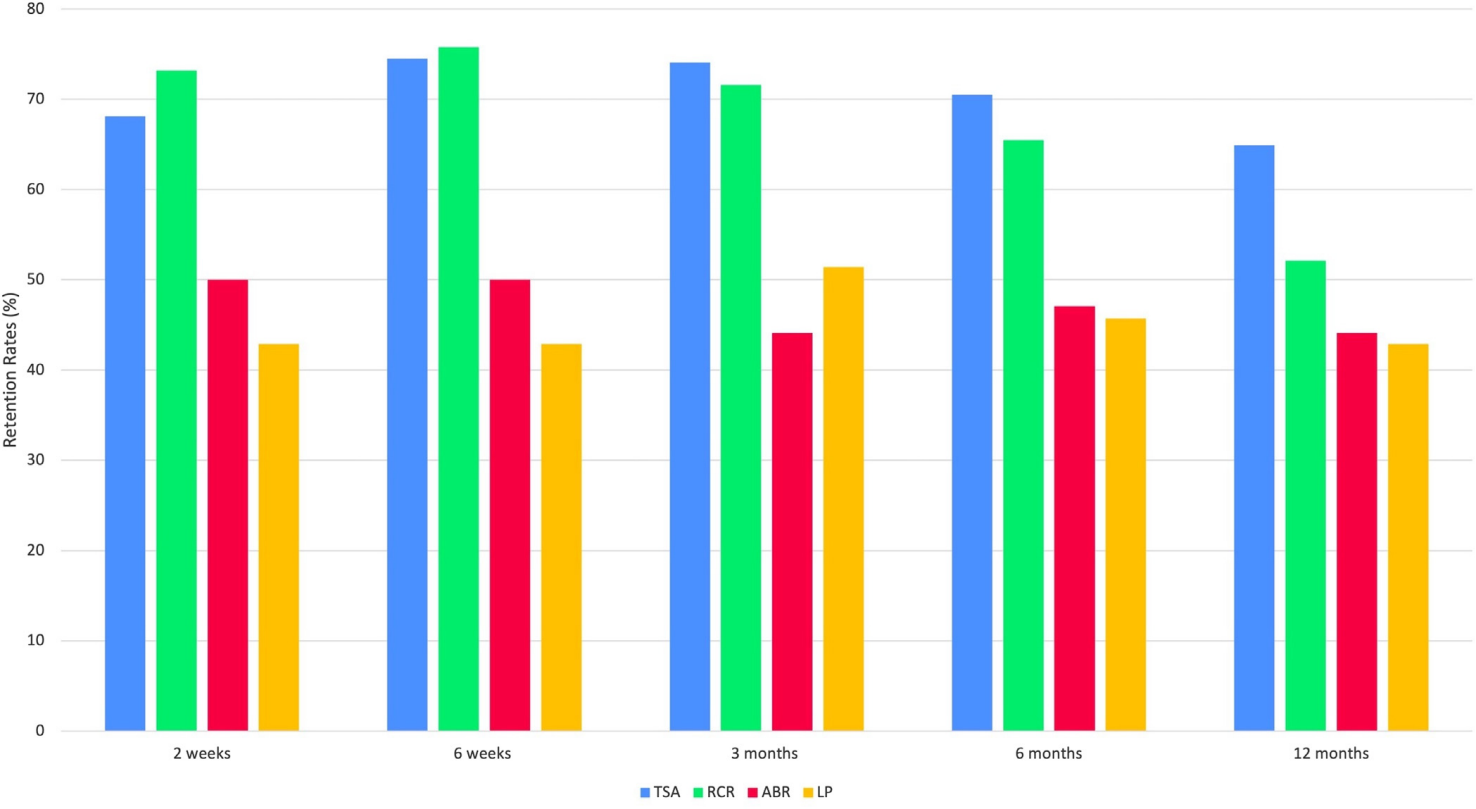
Retention rates by procedure.

The instability cohorts (LP and ABR) demonstrated a precipitous decline in their response rates (>50%) at first time point (2 weeks) but did not demonstrate much decline at subsequent time points [ABR (R^2^ = −0.62) and LP (R^2^ = +0.01) from 2 weeks to 12 months). In contrast, the RCR cohort maintained a good response rate (>70%) till 3 months but demonstrated a gradual decline to approximately 50% response rate at the one-year time point [ R^2^ = −0.76 from 2 weeks to 12 months]. The TSA cohort maintained a persistent response rate of more than 65% at all time points [R^2^ = −0.16 from 2 weeks to 12 months]. Procedure retention rates and their corresponding trend lines are shown in Figure 1.

### Analysis of factors predictive of response rate

Patient characteristics were compared between patients completing 12-month follow-up and those who failed to follow-up at 12 months. Patients who completed 12-month follow-up were older [61 ± 14 vs. 54 ± 17; *P* < .001], less frequently self-identified as Hispanic [13% vs. 23%; *P* = .009], less frequently single [32% vs. 44%; *P* = .008], and more frequently classified as ASA II [65% vs. 51%; *P* = .001]. A comprehensive comparison of patient characteristics between those completing and those failing to complete 12-month surveys can be seen in Table III.

**Table III:** Comparison of patient characteristics for completion of 12-month survey for all procedures.

	Overall (n = 514)	Incomplete (n = 178)	Complete (n = 336)	*P* value
Age, mean (SD)	58.6 (15.7)	54.3 (17.2)	60.8 (14.3)	<.001
Age group, n (%)				
<25	30 (5.8)	16 (9.0)	14 (4.2)	.043
25-35	27 (5.3)	17 (9.6)	10 (3.0)	.003
35-45	29 (5.6)	12 (6.7)	17 (5.1)	.558
45-55	71 (13.8)	30 (16.9)	41 (12.2)	.187
55-65	151 (29.3)	44 (24.7)	107 (31.8)	.129
65+	206 (40.1)	59 (33.1)	147 (43.8)	.025
Sex, n (%)				
Female	216 (42.0)	67 (37.6)	149 (44.3)	.170
Male	298 (58.0)	111 (62.4)	187 (55.7)	.170
BMI, mean (SD)	29.2 (6.1)	28.9 (5.5)	29.3 (6.4)	.541
Race, n (%)				
African American	49 (9.5)	14 (7.9)	35 (10.4)	.436
Asian	16 (3.1)	3 (1.7)	13 (3.9)	.276
Hispanic	84 (16.3)	40 (22.5)	44 (13.1)	.009
White	365 (71.0)	121 (68.0)	244 (72.6)	.317
Smoking status, n (%)				
Ever	215 (41.8)	84 (47.2)	131 (39.0)	.089
Never	299 (58.2)	94 (52.8)	205 (61.0)	.089
Marital status, n (%)				
Single	184 (35.8)	78 (43.8)	106 (31.5)	.008
Married	293 (57.0)	92 (51.7)	201 (59.8)	.093
Divorced	37 (7.2)	8 (4.5)	29 (8.6)	.122
ASA classification, n (%)				
ASA I	53 (10.3)	32 (18.0)	21 (6.2)	<.001
ASA II	310 (60.3)	92 (51.7)	218 (64.9)	.005
ASA III	146 (28.4)	50 (28.1)	96 (28.6)	.990
ASA IV	1 (0.2)		1 (0.3)	1.000
ASA unknown	4 (0.8)	4 (2.2)		.014
SDI score, n (%)				
1	94 (18.3)	32 (18.0)	62 (18.5)	.788
2	44 (8.6)	12 (6.7)	32 (9.5)	
3	101 (19.6)	30 (16.9)	71 (21.1)	
4	144 (28.0)	58 (32.6)	86 (25.6)	
5	56 (10.9)	18 (10.1)	38 (11.3)	
6	44 (8.6)	17 (9.6)	27 (8.0)	
7	23 (4.5)	8 (4.5)	15 (4.5)	
8	3 (0.6)	1 (0.6)	2 (0.6)	
9	0 (0.0)	0 (0.0)	0 (0.0)	
10	5 (1.0)	2 (1.1)	3 (0.9)	
Comorbidities				
DM, n (%)	73 (14.2)	19 (10.7)	54 (16.1)	.125
HTN, n (%)	260 (50.6)	88 (49.4)	172 (51.2)	.775
HLD, n (%)	235 (45.7)	70 (39.3)	165 (49.1)	.043

*SD*, standard deviation; *BMI*, body mass index; *SDI*, social deprivation index; *ASA*, American Society of Anesthesiology; *DM*, diabetes mellitus; *HTN*, hypertension; *HLD*, hyperlipidemia.

Multivariable logistic regression analysis was performed to identify factors predictive of completing the 12-month follow-up survey for all procedures (Table IV). Hispanic patients had lower odds of completing 12-month follow-up (odds ratio [OR]: 0.53, confidence interval [CI]: 0.32-0.88; *P* = .015) compared to non-Hispanic patients. When controlling for confounding factors including age, gender, race, and ASA status, the TSA cohort was more likely to complete 12-month surveys as compared to the LP (OR: 2.46, CI: 1.21-5.06, *P* = .013), ABR (OR: 2.34, CI: 1.13-4.84, *P* = .021), and RCR (OR: 1.71, CI: 1.16-2.50, *P* = .006) cohorts. RCR demonstrated a higher likelihood of 12-month completion compared to LP (*P* = .31) and ABR (*P* = .39); however, this did not reach significance. In turn, there were no differences in the likelihood of 12-month completion rates between other procedures (*P* > .05). Compared to the younger (23-34-year-old) patient cohort, patients who were older (65+ years) had a higher odds of completing the 12-month survey (OR: 3.21, CI: 1.40-7.31, *P* = .005).

**Table IV:** Univariate analysis factors predictive of completing 12-month follow-up for all procedures.

Variable	Odds ratio	95% confidence interval	*P* value∗
Age			
<25	Reference		
25-34	1.20	0.41-3.47	.730
35-44	2.55	0.87-7.42	.086
45-54	2.19	0.88-5.41	.088
55-64	2.29	0.99-5.29	.052
65+	3.21	1.40-7.31	.005
Gender			
Male	0.92	0.63-1.34	.686
Female	Reference		
Race			
African American	0.62	0.33-1.16	.136
Asian	2.24	0.69-7.19	.174
Hispanic	0.53	0.32-0.88	.015
White	Reference		
ASA classification			
I	Reference		
II	1.46	0.76-2.71	.217
III	1.44	0.74-2.78	.275
Smoking status			
Never smoker	Reference		
Ever smoker	0.96	0.66-2.42	.874
Marital status			
Married	1.31	0.88-1.93	.181
Not married	Reference		
Procedure			
LP	Reference		
RCR	1.44	0.70-2.99	.317
ABR	1.05	0.39-2.72	.915
TSA	2.46	1.21-5.06	.013
LP	0.69	0.33-1.42	.317
RCR	Reference		
ABR	0.72	0.34-1.51	.393
TSA	1.71	1.16-2.50	.006
LP	0.95	0.36-2.46	.915
RCR	1.37	0.66-2.86	.393
ABR	Reference		
TSA	2.34	1.13-4.84	.021
LP	0.40	0.19-0.83	.013
RCR	0.59	0.39-0.85	.006
ABR	0.42	0.21-0.87	.013
TSA	Reference		
Comorbidities			
DM	1.12	0.49-3.11	.650
HLD	1.24	0.84-1.82	.270
HTN	1.12	0.74-1.71	.571
BMI			
Normal weight	Reference		
Overweight	0.95	0.58-1.55	.581
Obese	0.86	0.52-1.43	.862
Social deprivation index			
<25th percentile	0.75	0.39-1.41	.375
25-50th percentile	0.49	0.28-0.89	.018
50-75th percentile	Reference		
75-100 percentile	0.34	0.07-1.67	.186

*ASA*, American Society of Anesthesiology; *LP*, Latarjet procedure; *RCR*, rotator cuff repair; *ABR*, arthroscopic Bankart repair; *TSA*, total shoulder arthroplasty; *DM*, diabetes mellitus; *HLD*, hyperlipidemia; *HTN*, hypertension; *BMI*, body mass index.

∗ *P* value in comparison to “Reference” for each variable.

## Discussion

The goal of this study was to determine if shoulder procedure type, as a denominator, influenced the one-year PROM survey completion rate among patients. We found that the overall survey completion rate at one year was approximately 57% for the four shoulder surgeries (TSA, RCR, ABR, and LP). There were unique differences among the different shoulder surgery cohorts regarding the rate and trend of attrition for PROM survey completion during the first year after surgery. Patients failing to complete 12-month surveys were younger (*P* < .001), single (*P* = .008), and Hispanic (*P* = .009). When controlling for confounding factors (age, gender, race, and ASA status), Hispanic ethnicity (*P* = .015) was associated with lower odds of completing the 12-month survey, while age >65 years old (*P* = .005) and the TSA procedure (*P* = .013) were associated with a higher odds of survey completion at 12 months.

Despite the administration of identical PROM surveys, our study demonstrated different survey completion rates between procedures at each time point (*P* < .05). Survey completion rates demonstrated early attrition to less than 50% in the ABR and LP cohorts at 2-weeks postoperatively but did not change considerably thereafter. The RCR cohort demonstrated an approximate 21% decline in completion rate throughout the study period with the greatest attrition at 6-months follow-up. The TSA cohort displayed the lowest attrition in response rate at one year with the highest 12-month completion rate (65%). One possible explanation for the sixth-month drop-off in RCR patients is a high degree of postoperative satisfaction.^10^^,^^11^^,^^14^^,^^25^^,^^26^^,^^33^^,^^34^^,^^52^ Additionally, the majority of patients after RCR, ABR, or Latarjet are back to their presurgery activity level by 6 months and may not feel the need to respond to surveys as they typically do not have subsequent in-person follow-ups. In contrast, patients after shoulder arthroplasty are typically seen at one-year follow-up and thereafter and may feel the need to continuously fill out PROM surveys. While Charousset et al and Kurowicki et al both reported postoperative improvement in these patients to plateau at one year postoperatively, there is nearly a 90% improvement in pain and function at six months postoperatively.^10^^,^^25^ Regarding TSA, the high response rates in these patients was mirrored in a study by Pines et al which established excellent responsiveness and correlation of P-UE to legacy outcome measures, finding 84.4% (81/96) follow-up in TSA patients at 12 months.^38^

For patients undergoing shoulder surgery for shoulder instability, we appreciate that over 74% of either instability cohort (LP and ABR) was under 35 years old, with a majority of the patients under 25 (40% of LP, 44.1% of ABR) when compared to the RCR and TSA cohorts. Consequently, we believe the poor retention rate to be most likely a product of a predominantly younger demographic, a previously ascertained risk factor for reduced survey response and follow-up.^7^^,^^11^^,^^14^^,^^16^^,^^28^^,^^33^^,^^52^^,^^54^^,^^55^ Conversely, looking at the TSA cohort with the highest response rate, we observe that 88.5% of patients are older than 55 years old, with 59.8% of the patients being over the age of 65 years old. As Ross et al demonstrated, those over 67 years old were more likely to express a desire to receive a survey by mail vs. email/phone.^43^ In a patient demographic that may be less technologically savvy or may not have access to such resources, the higher response rate of older patients in our study could be self-selecting as those without computer access may have felt discouraged from consenting to the study and only those who felt proficient participated. Moreover, it is possible that younger patients tend to have busier lifestyles with less time to respond which may have contributed to the poor survey completion rates observed in our study.^21^ Studies have postulated one possible etiology of differing response rates to be based on “inconvenience” and a patient's perceived level of satisfaction, whereby they no longer seek follow-up with their provider if they are satisfied with the outcome.^11^^,^^14^^,^^33^^,^^52^ When evaluating responders vs. nonresponders, Ross et al found that a greater percentage of nonresponders were satisfied with their procedure (87.1% vs. 83.8%; *P* = .480) compared to those who initially responded.^43^

When establishing patient-physician relationships, understanding cultural values and differences when assessing a patient's understanding is paramount. Several studies have demonstrated non-White race to be associated with lower completion rates following surgery.^3^^,^^36^^,^^45^ Interestingly, our study found Hispanic ethnicity to be associated with lower odds of survey completion at 12 months (*P* = .015). Hispanic ethnicity and lower survey completion rates may be partially attributable to language barriers. Despite our inclusion criteria necessitating that patients be proficient in English to consent for enrollment, further attention may need to be focused toward evaluating potential language barriers prior to enrollment. Poor communication leading to a misunderstanding regarding follow-up has also been postulated to impact the response rate of patients.^14^,^44^ In turn, physician distrust is a well-documented phenomenon in ethnic minorities which may contribute to lower completion rates.^4^^,^^9^ Unaccounted-for socioeconomic factors are important to consider, as well as the number of jobs, hours worked, and availability of auxiliary help at home, which may also contribute to lower completion rates in minority populations.^9^,^44^ For these reasons, we are unable to determine if the low completion rates in this population are attributable to other potential contributing factors.

Understanding ways to bolster research participation is integral to reducing disparities in health care. Clinician and administration engagement, OR: 19.6 (*P* < .001) and OR: 16.0 (*P* = .001), respectively, have been identified as the greatest two methods of increasing the collection rate of patient-reported outcomes.^47^ Providing monetary compensation for surveys has also been explored as an option for increasing responsiveness and has demonstrated positive results.^47^^,^^57^ In addition to the aforementioned methods of increasing responsiveness, we postulate that an increase in survey completion rates can also be attained by identifying the most vulnerable populations and clinical conditions associated with reduced responsiveness, as we have done through this study, and ensuring extra focus is placed upon collecting information from them by providers in office.

As with all studies, ours does not come without limitations. First, we did not offer the surveys via postal mail. Though this may have impacted older age group patients (>65 years), subjects were offered additional means (in-person, over the phone) to participate in the study which has shown no difference in outcome scores.^1^^,^^6^^,^^43^ Second, our study was limited to English-speaking participants, limiting the external validity of our study as translation of the PROMs would have required tests to demonstrate their validity was retained after translation.^41^ Third, multiple providers were involved in the study across our institution which introduced a level of variability of clinician engagement. As mentioned previously, clinical engagement has demonstrated a large impact on patient responsiveness, and we believe further studies can be done to determine the factors clinicians possess that demonstrate the highest level of patient survey completion.^47^ Thirdly, due to the nature of our survey process, we were unable to determine the total number of patients asked to participate; however, we aimed to determine the number and percentage of patients who completed surveys after being enrolled into our study. Lastly, our study was performed at a single institution in a metropolitan area which can introduce a limitation as geographic variance has demonstrated an impact on survey completion rates.^30^

## Conclusion

Postoperative PROM survey completion rates vary significantly among commonly performed shoulder procedures during the first year after surgery. Hispanic ethnicity and younger age were all predictive of a lower propensity, and the TSA procedure is predictive of higher odds for PROM survey completion at the 12-month follow-up.

## Disclaimers

Funding: No funding was disclosed by the authors.

Conflicts of interest: The authors, their immediate families, and any research foundation with which they are affiliated have not received any financial payments or other benefits from any commercial entity related to the subject of this article.
